# Linking molar organizational climate and strategic implementation climate to clinicians’ use of evidence-based psychotherapy techniques: cross-sectional and lagged analyses from a 2-year observational study

**DOI:** 10.1186/s13012-018-0781-2

**Published:** 2018-06-25

**Authors:** Nathaniel J. Williams, Mark G. Ehrhart, Gregory A. Aarons, Steven C. Marcus, Rinad S. Beidas

**Affiliations:** 1 School of Social Work, Boise State University, 1910 University Drive, Boise, ID 83725 USA; 2 Department of Psychology, University of Central Florida, Orlando, FL, USA; 3 Department of Psychiatry, University of California, San Diego, CA, USA; 4 School of Social Policy and Practice, University of Pennsylvania, Philadelphia, PA, USA; 5 Department of Psychiatry, University of Pennsylvania, Philadelphia, PA, USA

**Keywords:** Implementation climate, Molar climate, Organizational climate, Cognitive behavioral therapy, Behavioral health, Evidence-based practice, Moderation, Interactive effects

## Abstract

**Background:**

Behavioral health organizations are characterized by multiple organizational climates, including molar climate, which encompasses clinicians’ shared perceptions of how the work environment impacts their personal well-being, and strategic implementation climate, which includes clinicians’ shared perceptions of the extent to which evidence-based practice implementation is expected, supported, and rewarded by the organization. Theory suggests these climates have joint, cross-level effects on clinicians’ implementation of evidence-based practice and that these effects may be long term (i.e., up to 2 years); however, no empirical studies have tested these relationships. We hypothesize that molar climate moderates implementation climate’s concurrent and long-term relationships with clinicians’ use of evidence-based practice such that strategic implementation climate will have its most positive effects when it is accompanied by a positive molar climate.

**Methods:**

Hypotheses were tested using data collected from 235 clinicians in 20 behavioral health organizations. At baseline, clinicians reported on molar climate and implementation climate. At baseline and at a 2-year follow-up, all clinicians who were present in the organizations reported on their use of cognitive-behavioral psychotherapy techniques, an evidence-based practice for youth psychiatric disorders. Two-level mixed-effects regression models tested whether baseline molar climate and implementation climate interacted in predicting clinicians’ evidence-based practice use at baseline and at 2-year follow-up.

**Results:**

In organizations with more positive molar climates at baseline, higher levels of implementation climate predicted increased evidence-based practice use among clinicians who were present at baseline and among clinicians who were present in the organizations at 2-year follow-up; however, in organizations with less positive molar climates, implementation climate was not related to clinicians’ use of evidence-based practice at either time point.

**Conclusions:**

Optimizing clinicians’ implementation of evidence-based practice in behavioral health requires attention to both molar climate and strategic implementation climate. Strategies that focus exclusively on implementation climate may not be effective levers for behavior change if the organization does not also engender a positive molar climate. These findings have implications for the development of implementation theory and effective implementation strategies.

## Background

Evidence-based psychosocial interventions have been developed to effectively treat the most common psychiatric disorders of childhood and adolescence [1]; however, these interventions are seldom used and often poorly implemented in community behavioral health settings, contributing to the generally poor outcomes of these service systems [2–6]. Designing implementation strategies to improve the adoption, implementation, and sustainment of evidence-based practices (EBPs) in community settings requires an understanding of the determinants at multiple levels that influence clinicians’ practice behaviors as well as the dynamic ways in which those determinants interact [7]. Research testing potential determinants is underway [8]; however, the emerging literature is characterized by two important gaps. First, although conceptual models of implementation such as the Consolidated Framework for Implementation Research [9] and the Exploration, Preparation, Implementation, and Sustainment model [10] indicate that contextual characteristics of work environments interact in complex ways to influence clinicians’ implementation behavior, few empirical studies have developed theory or tested interactions between *general* organizational characteristics (e.g., molar organizational climate), which have a long research history and are common across many work environments, and *implementation-specific* organizational characteristics (e.g., implementation climate), which have an emerging research base and are believed to be most proximal to effective implementation of EBP [11]. This is a critical gap because understanding how these features of the work environment interact is necessary to generate effective implementation strategies and target strategies to contexts where they will be most effective. Second, few empirical studies have examined long-term relationships between organizational implementation determinants at baseline and subsequent implementation outcomes. As a result, minimal progress has been made in identifying organizational implementation determinants with long-term predictive validity or in testing or elaborating cross-level implementation theory [12].

This study addresses these gaps by examining how two types of organizational climate—molar organizational climate, which encompasses clinicians’ shared perceptions of how the work environment influences their personal well-being [13, 14], and strategic implementation climate, which includes clinicians’ shared perceptions of the extent to which the use of EBP is expected, supported, and rewarded by the organization [15–17]—interact in their concurrent and long-term relationships with clinicians’ implementation of EBP. Health and behavioral health organizations are characterized by multiple organizational climates (e.g., molar climate, implementation climate) which are hypothesized to have simultaneous, cross-level effects on clinicians’ EBP implementation [9, 10]. In this study, we propose and test an interactive model in which molar climate moderates implementation climate’s concurrent and long-term relationships with clinicians’ use of EBP. Understanding how multiple climates in organizations interact in their relationships with clinicians’ EBP implementation is critical to elucidating cross-level theory in implementation science, developing effective implementation strategies, and targeting strategies to settings where they will be most effective.

### Molar organizational climate

Molar organizational climate, defined as employees’ shared perceptions of the impact of the work environment on their personal well-being [14], has a long history of research in the organizational sciences and is highlighted by multiple implementation frameworks as a general (i.e., molar, non-implementation-specific) organizational characteristic that may influence clinicians’ EBP implementation [10, 13, 14, 18]. The concept of organizational climate refers to employees’ Gestalt perceptions of the work environment and incorporates numerous sub-dimensions (e.g., cooperation, support), which empirical studies have shown to represent a single, overarching factor that captures employees’ global evaluation of how the work environment impacts their personal well-being [14, 19]. In this study, we captured the molar climate perceptions clinicians share with regard to the well-established dimensions of cooperation and support from colleagues, opportunities for growth and advancement, and role clarity [14]. Clinicians develop shared molar climate perceptions as they experience their organization’s formal and informal policies, procedures, and practices and interpret how those experiences influence their personal well-being [20]. Researchers view molar climate as one dimension of an organization’s larger social context which may also include other more focused dimensions such as a strategic implementation climate [21].

Research on molar climate has linked these shared perceptions to broad indicators of improved service quality and outcomes in behavioral health services [18, 22–25]. However, empirical studies testing molar climate’s relationship with implementation outcomes in behavioral health have produced mixed results. More positive molar climates have been linked to improved clinician attitudes towards EBP, increased intentions to use EBP, and increased self-reported use of EBP [26–28]. However, other research has failed to link molar climate to clinicians’ self-reported use of EBP or to observer-coded clinician skill in EBP [29], and one study found that more positive molar climates predicted more negative clinician attitudes towards EBP and lower clinician knowledge of EBP [11]. These mixed findings highlight two gaps in our understanding of how molar climate relates to EBP implementation.

First, theory explaining how and why molar climate should be linked to EBP implementation has not been well articulated. Second, the inconsistent relationship between molar climate and implementation outcomes suggests the nature of the relationship between molar climate and implementation outcomes may be more complex than has typically been studied. Below, we address these issues by developing a theoretical formulation that explains why molar climate should act primarily as a *moderator* of the effect of implementation climate on clinicians’ EBP implementation.

### Implementation climate

Implementation climate is an organizational characteristic that refers to clinicians’ shared perceptions of the extent to which their organization expects, supports, and rewards the use of a specific innovation such as EBP [15–17]. As defined by Ehrhart et al. [16], implementation climate is the organizational characteristic that is most proximal to the implementation of EBP because it describes the extent to which clinicians perceive that their organization enacts a true priority for EBP implementation versus merely espousing such a priority [15–17, 30]. Clinicians develop shared perceptions of their organization’s implementation climate for EBP as they actively scan their work environment for cues regarding the specific behaviors that are most likely to be expected, supported, and rewarded (e.g., EBP use versus other behaviors) and develop shared interpretations of those cues through social interactions [21]. The most salient cues consist of organizational policies, procedures, and practices as well as formal and informal communications from organizational leaders and supervisors [31–34]. Implementation climate is hypothesized to increase clinicians’ use of EBP by (a) ensuring clinician skill in EBP (e.g., through training), (b) providing incentives for EBP use and disincentives for EBP avoidance, and (c) removing barriers to EBP use [15, 30].

Implementation climate has a robust theoretical foundation; however, few empirical studies have tested its relationship to clinicians’ use of EBP in health services, and findings to date have been equivocal. In cross-sectional studies, higher levels of implementation climate have been linked to determinants of implementation (i.e., increased clinician knowledge of EBP and more positive clinician attitudes towards EBP) [11]; however, implementation climate has not been consistently linked to clinicians’ *use* of EBP [28, 35]. Furthermore, we could not locate any studies testing the long-term relationship between implementation climate at baseline and clinicians’ subsequent use of EBP in health or behavioral health organizations. This is an important gap because theory suggests that once an implementation climate is established, it should have an ongoing, long-term influence on the behavior of clinicians, including those clinicians who subsequently enter the organization. We address these gaps by testing a model that describes molar climate as a moderator of both the concurrent and long-term effects of implementation climate on clinicians’ EBP use.

### Interactive effects of molar climate and implementation climate on clinicians’ EBP use

There is a need for theory describing how molar climate and implementation climate simultaneously relate to clinicians’ use of EBP and corroboration from empirical studies [34, 36]. We draw on organizational climate theory [34] to suggest that molar climate and implementation climate *interact* in their cross-level relationships with clinicians’ implementation of EBP. Specifically, we propose that implementation climate only has positive concurrent and long-term effects on clinicians’ EBP implementation when it is accompanied by a positive molar climate that reflects a foundation of support for clinician well-being; when high levels of implementation climate are not accompanied by a positive molar climate, clinicians will be less willing to engage in the strategically focused implementation behaviors prioritized by the organization.

We build these predictions around research showing that employees have stronger performance-reward outcome expectances and increased affective attachment to their organization when they perceive that the organizational climate supports their personal well-being [37–40]. By creating a positive molar climate, an organization primes its clinicians to enact work-related behaviors that are prioritized by the organization because of clinicians’ increased performance-reward outcome expectancies and increased affective attachment [41]. However, clinicians must determine which behaviors are most valued by the organization; a positive molar climate on its own does not communicate to clinicians what behaviors are most strategically valued by the organization. Therefore, organizations must also develop high levels of implementation climate which signal to clinicians the organization’s priority for implementing EBP and consequently direct clinicians towards enacting EBP implementation behaviors as one way to support the organization’s strategic priorities [42]. Having developed positive molar climate perceptions *and* a clear sense of the organization’s strategic priorities and expectations for EBP implementation (i.e., implementation climate perceptions), clinicians are likely to reciprocate by enacting EBP implementation behaviors. Based on this formulation, implementation climate should have its strongest effects on clinicians’ EBP use when it occurs alongside a positive molar climate that communicates support for clinicians’ well-being.

### Long-term effects of molar climate and implementation climate

The research described above indicates that molar climate and implementation climate should interact as they influence the EBP implementation behavior of clinicians who are present in an organization at a concurrent point in time. However, theory and prior research on organizational climate also suggests that implementation climate should have a stable, long-term (up to 2 years) relationship with the behavior of clinicians who remain in the organization after the organization’s climate has been established *as well as* with the behavior of clinicians who subsequently enter the organization [21]. From a theoretical standpoint, the effects of climate are expected to be long-lasting because they reflect the policies, practices, procedures, and systems in the organization, which are not necessarily easy to change. Even if an organization changes its policies and procedures such that they no longer communicate a priority for EBP implementation, it takes time for clinicians to recognize these changes, become convinced that they are lasting changes rather than temporary, and begin to change their behavior in response [21]. In addition, implementation climate should exhibit long-term effects on the behavior of employees who enter the organization after the climate has been established because climate is reinforced through processes of new employee socialization, clinicians’ social interactions with each other, and clinician attraction, selection, and attrition [43]. Thus, climate is perpetuated across time and is expected to have a stable long-term relationship with clinician behavior despite turnover in the clinician ranks.

Consistent with this theory, prior research has shown that implementation climate as measured at baseline predicts the implementation behavior of all employees who are present in manufacturing organizations up to 2 years later [31]. However, we could locate no studies in health or behavioral health services that tested the long-term relationship between baseline implementation climate and clinicians’ subsequent use of EBP. Furthermore, if our proposed interactive model holds for the concurrent relationship between implementation climate and clinicians’ use of EBP, it should also characterize the long-term relationship between implementation climate and clinicians’ use of EBP. That is, molar climate should moderate both the concurrent and long-term effects of implementation climate on clinicians’ use of EBP such that implementation climate has its strongest effects when it is accompanied by a positive molar climate. In this study, we provide the first test of these hypotheses.

### Study hypotheses

Hypotheses 1 and 2 test the concurrent (i.e., cross-sectional) relationships between implementation climate, molar climate, and the use of EBP by clinicians who are present at time 1. Hypothesis 1 states that higher levels of implementation climate at time 1 will predict increased clinician use of EBP at time 1. Hypothesis 2 states that the concurrent relationship between implementation climate at time 1 and clinicians’ use of EBP at time 1 will be moderated by molar climate at time 1 such that implementation climate will be more positively related to clinician EBP use when molar climate is high than when molar climate is low. These analyses test whether organizational climate is related to the concurrent behavior of clinicians who are present at time 1.

Hypotheses 3 and 4 test the long-term (i.e., 2-year lagged) relationships between implementation climate and molar climate at time 1, and the use of EBP by clinicians who are present in the organizations at time 2. These analyses test whether climate has a stable, long-term relationship with clinicians’ behavior; that is, they do not examine change in EBP use over time but rather if the level of climate in an organization at baseline continued to be associated with a certain level of clinician behavior at 2-year follow-up. Hypothesis 3 states that higher levels of implementation climate at time 1 will predict increased clinician use of EBP 2 years later at time 2. Hypothesis 4 states that the long-term relationship between implementation climate at time 1 and clinicians’ use of EBP at time 2 will be moderated by molar climate at time 1 such that implementation climate will be more positively related to EBP use when molar climate is high than when molar climate is low. These analyses test whether time 1 molar climate and implementation climate are related to the subsequent behavior of clinicians who remained in the organizations 2 years later at time 2 or who subsequently entered the organizations in the 2 years between time 1 and time 2. Together, the four hypotheses test climate theory by examining whether molar climate and implementation climate are characteristics of organizations that interact to influence the concurrent behavior of clinicians (i.e., cross-sectional analyses) as well as the subsequent behavior of clinicians (i.e., lagged analyses).

## Methods

### Setting

We conducted a prospective observational study of two types of organizational climate and clinicians’ EBP use in mental health organizations within the context of a system-wide initiative to support EBP implementation in a major northeastern city in the USA [44]. Details of the overarching policy initiative are provided elsewhere [45]. Briefly, since 2007, the Department of Behavioral Health and Intellectual DisAbility Services (DBHIDS) in the City of Philadelphia has sought to increase EBP implementation in the public behavioral health system by providing funds for training, expert consultation, and ongoing technical assistance to over 50 organizations in the Philadelphia system. These activities include promoting EBP among various stakeholder groups (e.g., leaders of organizations, clinicians, and consumers of mental health services), delivering training in EBP, providing organizations with technical assistance to implement EBP, and providing an enhanced reimbursement rate for delivery of targeted EBPs [45]. To date, DBHIDS has supported implementation of four specific evidence-based psychotherapy protocols based on principles of cognitive behavioral therapy which has extensive empirical evidence supporting its effectiveness in addressing youth psychiatric disorders [1]. A full-time city employee coordinates implementation, training, and consultation by treatment developers.

The creation of a uniform policy environment that supported EBP implementation within this system presented an ideal opportunity to study how variation in organizations’ molar climates and implementation climates related to clinicians’ EBP implementation amidst the context of an overarching EBP policy initiative. Furthermore, because the initiative extended over multiple years, it provided an opportunity to examine the lagged relationships between these organizations’ climates at baseline and the subsequent use of EBPs by clinicians who were present in the organizations 2 years later.

### Participants and procedures

Twenty-nine of the largest child-serving community behavioral health organizations within the Philadelphia metropolitan area, which serve approximately 80% of youth receiving publicly funded behavioral health care, were invited to participate. Of those 29 organizations, 18 agreed to participate (62%). Additionally, one organization implementing one of the four EBPs requested to participate, resulting in a final sample of 19 organizations at baseline. Given that some of these organizations had multiple sites with distinct locations and leadership structures, we included each site as a distinct organization (*k* = 23). Twenty of these organizations (87%) were retained at the 2-year follow-up resulting in a total sample of 20 organizations which were included in our analyses. Because we were interested in how organizational climate influenced the behavior of all clinicians who were present at baseline and at the 2-year follow-up, we invited all clinicians who provided direct mental health services to youth and their families and who were present in the organizations at either time point to participate in the study.

At baseline, 112 clinicians participated (overall response rate = 46%), and at 2-year follow-up 164 clinicians participated (overall response rate = 65%). The mean within-organization response rate across waves was 59% (min = 23%, max = 92%), and the mean number of clinicians per organization was 11.75 (min = 3, max = 39). Because of clinician attrition and new hires over the 2-year period, 41 participating clinicians (17.4%) were represented in both waves of data. Consistent with methodologists’ recommendations, we included all organizations with three or more respondents in our analyses regardless of response rates in order to improve the accuracy of statistical inferences and optimize statistical power [46, 47]. Similar to national samples [48], most clinicians in this study were female (77%); had graduate degrees at the master’s (84%) or doctoral (9%) levels in social work, psychology, or an allied health field; and were highly experienced in delivering behavioral health services (*M* = 9.17 years). Average clinician tenure in the organization was 3.04 years, and average age was 38.53 years. Nearly half the clinicians in the sample (42%) identified their primary theoretical orientation as cognitive-behavioral.

Data were collected in 2013 (time 1) and 2015 (time 2). We selected a 2-year follow-up period based on prior research [31, 49]. Clinicians completed study questionnaires on-site during regular work hours without clinical or work supervisors present in order to reduce demand characteristics and assure confidentiality. During a 1.5-h meeting at each organization, the principal investigator and trained research assistants provided lunch, obtained written informed consent, and administered questionnaires. At time 1, clinicians completed questionnaires addressing molar climate, implementation climate, their own use of psychotherapy techniques, and demographics. At time 2, clinicians provided data on their own use of psychotherapy techniques and demographics. Organizational leadership (e.g., clinical director, executive director) reported on organization size. All participants received $50 for study participation. All study procedures were approved by the City of Philadelphia Institutional Review Board and the University of Pennsylvania Institutional Review Board.

### Measures

#### Molar organizational climate

Molar organizational climate was measured using the 15-item *functionality* scale of the Organizational Social Context (OSC) measure [50]. The OSC was developed to assess organizational climate in public sector allied health settings and has demonstrated structural validity in two national samples [50, 51] as well as criterion-related validity [24, 25, 27, 28, 44, 52]. The functionality scale incorporates three first-order subscales that assess clinicians’ perceptions of cooperation and support from colleagues, opportunities for growth and advancement in the organization, and the extent to which they have a clear understanding of their role in the organization [50]. Confirmatory factor analyses indicate that the three subscales load on a single latent factor represented by the functionality total score [50, 51]. Item responses are made on a 5-point scale ranging from 1 (*Never*) to 5 (*Always*). Coefficient alpha for this scale was *α* = .92 in the present study.

#### Implementation climate

Clinicians’ perceptions of implementation climate for EBP were measured using the 18-item Implementation Climate Scale (ICS) [16]. Responses were made on a 0 (*Not at All*) to 4 (*A Very Great Extent*) response scale. The ICS total score is derived from items addressing six sub-dimensions including organizational focus on EBP, educational support for EBP, recognition for EBP, reward for EBP, selection for EBP, and selection for openness. Because of our interest in the overall EBP implementation climate rather than the sub-dimensions, we calculated the mean score across the dimensions for our analyses. In prior research, the overall ICS demonstrated excellent scale reliability (*α* = .91) and structural validity as well as convergent and discriminant validity [16]. The coefficient alpha for this scale was *α* = .94 in the present study.

#### Clinician EBP use

Clinicians’ self-reported use of EBP in their practice with clients was measured using the 33-item cognitive-behavioral subscale of the Therapy Procedures Checklist-Family Revised (TPC-FR) [53]. This measure asks clinicians to consider a specific client they are currently treating that is representative of their larger caseload and to endorse psychotherapy techniques they use with that client from a specified list. Items describing psychotherapy techniques represent three well-established schools of psychotherapy including cognitive-behavioral therapy, family therapy, and psychodynamic therapy. Clinicians could endorse techniques from all three treatment models using a continuum ranging from 1 (*rarely*) to 5 (*most of the time*). Because of the strong empirical support for cognitive behavioral therapy and our interest in examining EBP use [54], we used the cognitive behavioral subscale of the TPC-FR as the criterion variable in this study. Prior studies support the measure’s test-retest reliability, sensitivity to change, and criterion-related validity [53, 55]. Coefficient alphas for this scale at time 1 and time 2 were *α* = .94 and *α* = .92, respectively.

#### Control variables

To eliminate potential confounds and optimize statistical power, we included organization- and clinician-level covariates as control variables in the models testing our hypotheses. At the organization level, we included *size*, measured as the total number of full-time clinicians as reported by organization leadership, because prior research has linked size to climate effects [56, 57]. At the clinician level, we included average *hours worked per week* (as reported by clinicians), because clinicians with increased client contact hours may have increased clinical and documentation demands that contribute to less EBP use; clinicians’ *tenure with the organization*, because differential clinician exposure to an organization’s climate may influence its effects on employee behavior; and clinicians’ *education level* (i.e., doctoral level vs. less advanced degrees), because advanced graduate degrees may be associated with more training in EBPs [34]. In addition, we included clinicians’ primary *theoretical orientation* (i.e., CBT vs. non-CBT) and *attitudes towards EBP* (measured using the Evidence-Based Practice Attitude Scale [27]) as covariates in our models because these factors may influence clinicians’ use of CBT techniques.

#### Data aggregation

In order to ensure proper alignment between the levels of theory and analysis, we calculated organization-level values for molar climate and implementation climate by aggregating (i.e., averaging) clinicians’ individual responses to these scales. Evidence of within-organization agreement was provided by calculation of *r*_wg(j)_ values with a uniform null distribution; these values were all above the generally accepted .70 cutoff (range = .79 to .98) [58, 59]. Evidence of significant between-organization variance on these constructs was provided by analyses of variance (ANOVAs) with organization as the independent variable and clinicians’ individual-level climate responses as the dependent variable (all *p*s < .001, *η*^2^ = .41 for implementation climate, *η*^2^ = .35 for organizational climate). Taken together, these analyses demonstrated sufficient within-organization agreement and between-organization variance to support the aggregation of molar climate and implementation climate scores to the organizational level.

### Data analytic approach

Given the hierarchical structure of the data (i.e., clinicians nested within organizations) and our cross-level theoretical model, study hypotheses were tested using two-level mixed effects regression models in Mplus software version 8 [60, 61]. The models incorporated random organization intercepts and fixed slopes for clinician-level covariates given evidence that slopes did not differ across organizations (all *p*s > .3). All covariates were grand mean centered to facilitate model interpretation and to adjust models for between-organization differences in clinician composition [62]. Missing data for predictor variables was minimal (< 5%) and was addressed via maximum likelihood estimation [60].

Prior to calculating the interaction term for molar climate and implementation climate, these variables were centered to facilitate interpretation [63, 64]. The interaction between molar climate and implementation climate was probed using simple slopes analysis which tested the relationship between the focal predictor (implementation climate) and the outcome (clinician EBP use) at *high* and *low* values (± 1.5 standard deviations) of the moderator (molar climate) [64]. The first set of models (i.e., cross-sectional analyses) tested how molar climate, implementation climate, and their interaction related to the EBP use of all clinicians who were present at time 1. The second set of models (i.e., lagged analyses) tested how molar climate, implementation climate, and their interaction as measured at time 1 related to the subsequent EBP use of all clinicians who were present in the organizations at time 2.

Effect sizes were calculated by converting beta coefficients to standardized units and by calculating a pseudo incremental *R*^*2*^ based on the reduction in variance method described by Raudenbush and Bryk [61]. Preliminary analyses supported our multilevel approach by providing evidence of significant organization level variance in clinicians’ EBP use at time 1 (ICC(1) = .29, *p* < .001) and time 2 (ICC(1) = .12, *p* = .002).

## Results

### Cross-sectional relations between molar climate, implementation climate, and clinicians’ EBP use

Table 1 presents the mixed effects regression models testing our study hypotheses. Hypothesis 1 stated that higher levels of implementation climate at time 1 would predict increased clinician use of EBP at time 1. Results supported this hypothesis. Controlling for clinician and organization covariates and molar organizational climate, higher levels of implementation climate predicted increased clinician use of EBP (*B* = .48, SE = .24, *p* = .044) explaining 23% of the organization intercept variance over and above the other predictors (i.e., *R*^2^_incremental_ = .23). Controlling for the other variables, every one standard deviation increase in implementation climate predicted a .35 standard deviation increase in clinicians’ expected use of EBP.

**Table 1:** Two-level mixed effects regression models linking implementation climate and molar climate to clinicians’ EBP use

	Clinician EBP use			
	Cross-sectional analysis (baseline)		Lagged analysis (2-year follow-up)	
Predictor	*B* (SE)	*B* (SE)	*B* (SE)	*B* (SE)
Intercept	3.175 (.079)**	3.060 (.071)**	3.286 (.056)**	3.213 (.063)**
Tenure in organization	.024 (.012)	.025 (.012)*	.019 (.012)	.020 (.012)
Average hours per week	.007 (.005)	.007 (.004)	.008 (.004)	.008 (.003)*
Education level (doctoral)	.071 (.231)	− .014 (.229)	− .021 (.157)	− .094 (.176)
CBT theoretical orientation	.118 (.127)	.187 (.122)	.075 (.079)	.093 (.090)
Attitudes towards EBP	.019 (.113)	− .025 (.116)	.143 (.100)	.136 (.099)
Organization size (# of therapists)	.017 (.007)*	.012 (.005)*	.006 (.004)	.003 (.004)
Molar climate (baseline)	− .012 (.008)	.001 (.008)	− .006 (.006)	.002 (.007)
Implementation climate (baseline)	.480 (.239)*	.154 (.217)	.301 (.131)*	.016 (.198)
Molar climate x implementation climate		.021 (.007)**		.012 (.005)*
*Pseudo* model *R*^2^	.63	.98	.94	.98

Note: These are two-level mixed-effects regression models with random organization intercepts. *CBT* cognitive-behavioral therapy, *EBP* evidence-based practice. For the cross-sectional analysis, *k* = 20 organizations and *n* = 112 clinicians; for the lagged analysis, *k* = 20 organizations and *n* = 164 clinicians. *Pseudo* model *R*^2^ calculated as (*τ*_null_ − *τ*_model_)/(*τ*_null_) where *τ*_null_ is the organizational intercept variance in a model with no predictors and *τ*_model_ is the residual organizational intercept variance in a model including all predictors [61]

*p ≤ .05, **p < .01

**Fig. 1:**
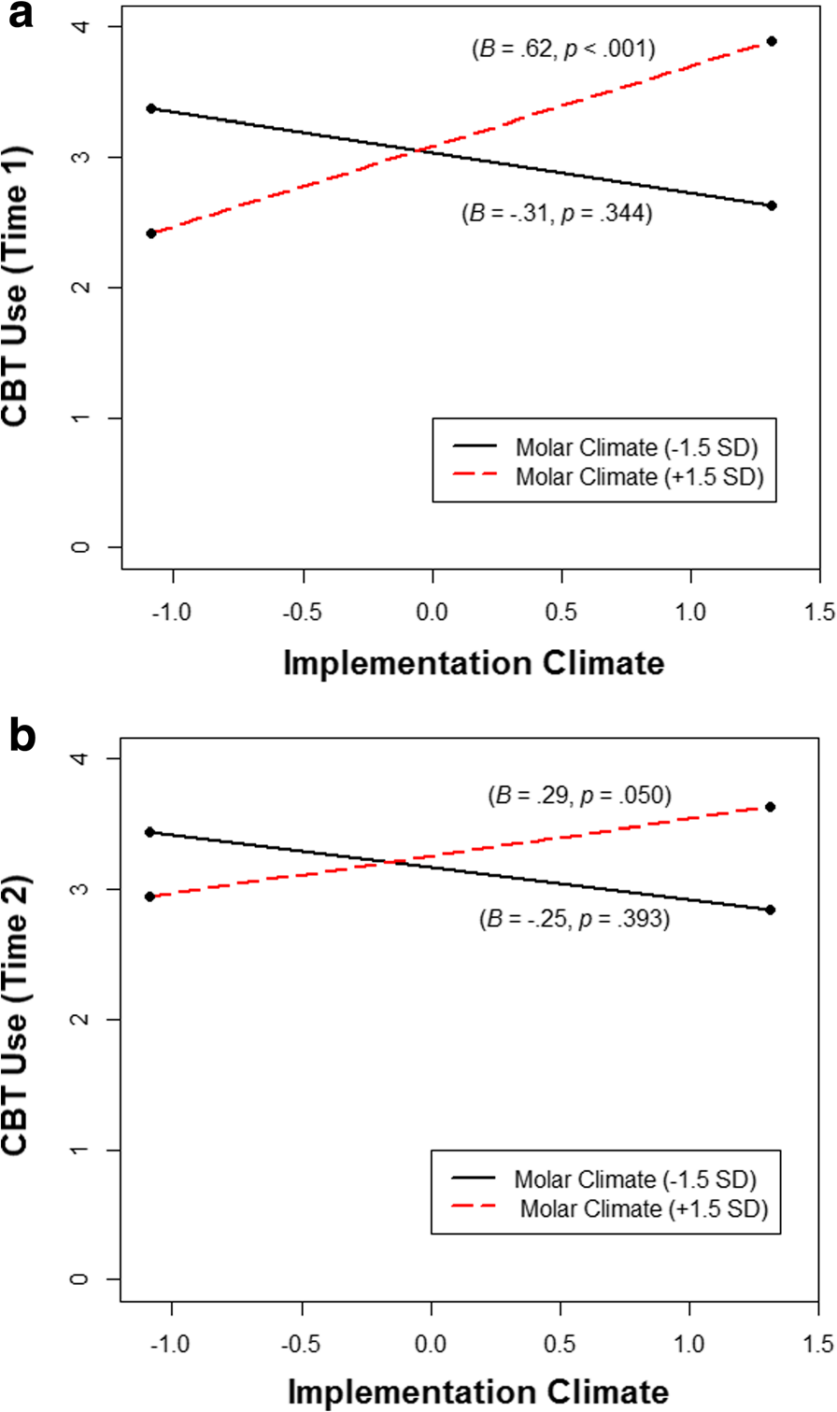
Interaction between baseline implementation climate and molar climate in predicting clinicians’ use of evidence-based practices. Note: These graphs show the statistically significant interactions between baseline implementation climate and molar climate in predicting clinicians’ use of cognitive behavioral psychotherapy techniques (CBT) at baseline (time 1) (**a**) and two-year follow-up (time 2) (**b**). Betas and *p* values reported in the figure represent simple slopes at conditional values of molar climate (± 1.5 SDs from the mean). Graphs are based on the results of two-level mixed-effects regression models with random organization intercepts. For the time 1 analysis, *k* = 20 organizations and *n* = 112 clinicians; for the time 2 analysis, *k* = 20 organizations and *n* = 164 clinicians

Hypothesis 2 stated that molar climate would moderate the cross-sectional relationship between implementation climate at time 1 and clinicians’ use of EBP at time 1 such that the relationship would be significantly more positive when molar climate was high than when molar climate was low. Results supported hypothesis 2 (see Table 1). The interaction between molar climate and implementation climate was significant in predicting clinicians’ use of EBP at time 1 (*B* = .02, SE = .01, *p* = .002) and accounted for an additional 35% of the organizational intercept variance (i.e., *R*^2^_incremental_ = .35). Most importantly, as is shown in Fig. 1a, the shape of the interaction was consistent with hypothesis 2. Higher levels of implementation climate predicted increased clinician use of EBP in organizations with more positive molar climates (*B* = .62, SE = .18, *p* < .001); specifically, for every one standard deviation increase in implementation climate, clinicians’ expected use of EBP at time 1 increased by .45 standard deviations. However, in negative molar climates, implementation climate was not predictive of clinicians’ use of EBP (*B* = −.31, SE = .33, *p* = .344). These findings support our conditional model of molar and implementation climate.

### Two-year lagged relations between molar climate, implementation climate, and clinicians’ EBP use

Hypothesis 3 stated that higher levels of implementation climate in the organizations at time 1 would predict increased clinician use of EBP 2 years later at time 2 in a lagged analysis. Results of the mixed-effects regression model supported this hypothesis. Higher levels of implementation climate at time 1 predicted increased use of EBP among clinicians who were present in the organizations 2 years later (*B* = .30, SE = .13, *p* = .022), accounting for 58% of the organizational intercept variance beyond that accounted for by the other variables. For every one standard deviation increase in implementation climate at time 1, clinicians’ expected use of EBP in these organizations at time 2 increased by .24 standard deviations.

Hypothesis 4 stated that the long-term relationship between implementation climate at time 1 and clinicians’ use of EBP in these organizations at time 2 would be moderated by molar climate at time 1 such that implementation climate would be more positively related to clinicians’ use of EBP when molar climate was high than when molar climate was low. Results from the lagged analysis supported hypothesis 4 (see Table 1). There was a significant interaction between baseline implementation climate and molar climate in predicting clinicians’ use of EBP in these organizations at the 2-year follow-up (*B* = .01, SE = .005, *p* = .028), and the shape of this interaction was nearly identical to the cross-sectional analysis (see Fig. 1b). Simple slopes analysis confirmed that when time 1 molar climate was high, time 1 implementation climate had a significant and positive relationship with EBP use among clinicians who were present in the organizations at time 2 (*B* = .29, SE = .15, *p* = .050); however, when time 1 molar climate was low, time 1 implementation climate was not related to clinicians’ use of EBP at time 2 (*B* = −.25, SE = .29, *p* = .393). In positive molar climates, every one standard deviation increase in implementation climate at time 1 predicted a .23 standard deviation increase in clinicians’ expected use of EBP in these organizations at time 2. Most importantly, the highest levels of EBP use occurred in both analyses when baseline molar climate was positive and baseline implementation climate was high (see Fig. 1a, b,).

## Discussion

The goals of this study were to build implementation theory and to inform implementation practice by testing a conditional model in which molar organizational climate moderated the concurrent and long-term relationships between implementation climate and clinicians’ use of EBP in behavioral health organizations. Results supported our hypotheses. In organizations with more positive molar climates at baseline, higher levels of implementation climate predicted increased EBP use among clinicians who were present at baseline (time 1) and among clinicians who were present in the organizations 2 years later (time 2); however, in organizations with less supportive molar climates at baseline, implementation climate was not related to clinicians’ use of EBP at either time point. This study advances implementation theory, research, and practice by showing that the general organizational characteristic of molar climate and the implementation-specific characteristic of implementation climate are *both* essential to supporting clinicians’ EBP implementation.

Results from this study help clarify the equivocal findings of prior research on climate and implementation in health and behavioral health services [11, 26–29, 35] by showing that molar climate and implementation climate are conditionally related; both must be present in an organization in order to support EBP implementation. In studies that have found a positive relationship between either type of climate and EBP implementation, it is likely that the other type of climate was also present; conversely, in studies that failed to link either type of climate to implementation, this null finding may be explained by a low level of the other type of climate. These findings also help clarify some of the complex relationships hypothesized by implementation frameworks [9, 10]. Specifically, our finding of an interactive effect suggests higher levels of complexity than models suggesting that molar climate and implementation climate have simultaneous but independent main effects on EBP implementation [11], and also suggests an alternative conceptualization to models that present molar climate as an antecedent to implementation climate [34, 36]. Finally, these results empirically confirm the proposed role of implementation climate suggested by implementation science frameworks [10] and by the organizational sciences literature [15] and also identify a boundary condition (i.e., positive molar climates) under which implementation climate has its strongest effects.

Findings from this study also provide important insights for developing implementation strategies, conducting pre-implementation assessments, and targeting strategies to settings where they will be optimally effective. Results suggest health and behavioral health organizations can optimize clinicians’ use of EBP by *simultaneously* developing implementation climates that expect, support, and reward clinicians’ use of EBP and by fostering positive molar climates that support clinicians’ well-being. The moderating effect of molar climate on implementation climate’s relationship with clinicians’ use of EBP suggests leadership must attend to the influence of the work environment on the well-being of clinicians in addition to the strategic issues of communicating a priority for EBP implementation. Theory and research on climate embedding mechanisms provide a useful framework for helping organizational leaders consider how they might develop a positive molar climate and a strong implementation climate within their setting [32, 65]. This research suggests that leaders contribute to climate through a number of actions that communicate their priorities and expectations. Leader behaviors that influence molar climate include how decisions are made in the organization (e.g., top down or with input from the front line), how mistakes and errors are handled, and the type of interpersonal style that is modeled [65]. Leader behaviors that support a strong implementation climate include paying attention to and measuring EBP implementation on a regular basis, allocating resources to support implementation, allocating rewards and status to clinicians who develop or maintain expertise in EBP, and providing role modeling or coaching in EBP [32]. By focusing on the dimensions of implementation climate deemed most likely to support clinical practice change in their particular setting, and by ensuring that clinicians experience positive cooperation and support from colleagues and supervisors, opportunities for growth and advancement, and role clarity, organizational leaders can optimize clinicians’ EBP implementation.

A further implication of these findings is that the outcomes of implementation efforts may be enhanced by conducting pre-implementation assessments of molar organizational climate and implementation climate. To the extent that an organization does not engender a positive molar climate, this suggests that preliminary work to enhance the organization’s molar climate might be indicated prior to expending resources on a strategy to develop a strong implementation climate. Furthermore, implementation initiatives that focus exclusively on implementation climate and ignore clinicians’ sense of well-being in the organization may not achieve positive results. Although strategies that address both the general organizational characteristic of molar climate and the implementation-specific characteristic of implementation climate may be more time or resource intensive than strategies that focus on implementation climate alone, results from this study suggest the added resources may be worth the expense and potentially necessary to realize the implementation strategy’s intended effects [66], especially when the molar climate levels are low. Tools for assessing molar climate and implementation climate include the organizational social context measure developed by Glisson and colleagues [50] and the implementation climate scale developed by Ehrhart et al. [16].

The finding that baseline molar climate moderated the long-term relationship between implementation climate at baseline and clinicians’ use of EBP in these organizations 2 years later is consistent with climate theory and research in manufacturing which has shown similar relationships between strategic climates (e.g., implementation climate, service climate, safety climate) and strategic outcomes (e.g., innovation implementation, customer service, manufacturing accidents) over a 2-year time lag [31, 49]. Although climate is generally viewed as more malleable than organizational culture [21], there have been differing views in the literature with regard to how easily climate can be changed. Findings from this study are consistent with the perspective that although the policies and procedures that are the foundation of climate may be easily changed, changing employees’ perceptions of those policies and procedures and the meanings associated with them does not happen quickly [21, 67]. In combination with other studies showing climate’s predictive validity across a 2-year time lag [31, 49], these findings suggest that implementation climate has long-term predictive value for forecasting clinicians’ use of EBP.

The present study demonstrates that there is a need for more nuance in how we consider implementation frameworks to theorize and test the ways in which organizational context may influence adoption, implementation, and sustainment of EBPs in usual care and public sector settings [10]. The particular question of the relative or synergistic influence of molar and strategic climates begs the question of how system and organization leaders set the stage and develop positive climates. Work on leadership development is ongoing in identifying specific leader characteristics and behaviors that, in tandem with targeted organizational strategies, may improve organizational and implementation climate [68]. The present study examined some aspects of inner organizational context within the outer context of a broad system change initiative. Consistent with this, recent work has demonstrated ways in which leaders at both system and organizational levels can set the stage and support EBP implementation and sustainment [69]. This approach has potential to improve EBP quality, fidelity, and public health outcomes.

### Study strengths and limitations

This study has a number of strengths and limitations. First, molar climate and implementation climate were not experimentally manipulated. Thus, our results are correlational and do not support causal inferences. Furthermore, the lagged analyses in this study were not designed to examine change in EBP use over time and therefore do not provide a basis for making inferences regarding whether a change in organizational climate contributes to a change in clinicians’ EBP use. Although these analyses show that organization-level implementation climate and molar climate preceded the use of EBP by clinicians in these organizations 2 years later, experimental studies are needed to examine whether change in these climates contributes to change in clinicians’ EBP use and can therefore act as a mechanism to change clinicians’ behavior [70].

Second, clinicians in our study provided information on both the organizational climate and their use of EBP, raising the possibility of common method variance confounding our study results [71, 72]. These concerns are mitigated, however, by four factors. First, the interaction effects observed in this study could not have been caused by common method variance [73]. Second, temporal separation of measurement is one strategy to mitigate possible common method variance [71], and the replication of the hypothesized effects in cross-sectional and lagged analyses provides increased confidence in their validity. Third, with regard to organizational climate, the use of self-report measures provides an optimal way to assess clinicians’ *perceptions* of the organization (as opposed to the objective organizational reality) and to test the extent to which those perceptions are shared. This is important because clinicians respond to their *perceptions* of an organization’s climate rather than to the objective conditions within the organization and because it is necessary to demonstrate that those perceptions are shared in order to argue that the climate is a characteristic of the organization instead of the individual [21]. Fourth, because the climate perceptions are combined with other employees’ perceptions in the aggregation process, the potential for possible bias in the relationships found with EBP use is further mitigated.

Third, we relied on clinicians’ reports of psychotherapy technique use based on a standardized self-report. Although clinicians are uniquely privy to their in-session behaviors and, in particular, to the types of techniques they *try* to use in sessions, clinician reports do not always demonstrate strong concordance with observational measures [74]. However, confidence in the reliability of clinicians’ reports in the present study is increased by three factors. First, the measure of EBP use and the procedures used to collect the measures reduced concerns about social desirability biases by providing a full spectrum of techniques from which clinicians could choose across three well-established schools of psychotherapy and by ensuring confidentiality of responses so that leadership and peers were unaware of how clinicians responded. Second, the measure facilitated report accuracy by prompting clinicians to identify a specific client for their responses and by providing an exhaustive list of highly specific descriptions of widely recognized techniques. Third, the wide range of values and the normal distribution of scores observed on the EBP use measure suggest clinicians’ reports were not unduly skewed in a specific direction. These features increase confidence in the veracity and reliability of clinicians’ reports.

Fourth, we measured clinicians’ use of cognitive behavioral therapy because of its strong empirical support [1] and because the larger policy environment in which these organizations were embedded was focused on increasing the use of this therapeutic approach. There are other EBPs for youth psychiatric disorders, however, and our findings may not generalize to other treatments, particularly if those treatments are not as heavily influenced by organizational factors. This concern is mitigated to some extent by the fact that a large proportion of EBPs for youth psychiatric disorders incorporate cognitive behavioral procedures [54].

## Conclusions

This study advances implementation theory and practice by demonstrating that optimal EBP implementation occurs in organizations with both positive molar climates and high levels of strategic implementation climate. In support of international priorities to increase the delivery of EBP in health and behavioral health settings, these findings suggest organizations can facilitate the delivery of effective treatments by developing organizational climates that engender high expectations and support for EBP implementation as well as strong support for clinician well-being. Further, this work advances implementation research by elucidating the relationships between determinants of common implementation science frameworks and implementation outcomes and presenting targets for implementation strategies.

## Abbreviation

EBP: Evidence-based practice
